# Predictive model of the dropout intention of Chilean university students

**DOI:** 10.3389/fpsyg.2022.893894

**Published:** 2023-01-05

**Authors:** Yaranay López-Angulo, Fabiola Sáez-Delgado, Javier Mella-Norambuena, Ana B. Bernardo, Alejandro Díaz-Mujica

**Affiliations:** ^1^Departamento de Psicología, Facultad de Ciencias Sociales, Universidad de Concepción, Concepción, Chile; ^2^Centro de Investigación en Educación y Desarrollo (CIEDE-UCSC), Departamento Fundamentos de la Pedagogía, Facultad de Educación, Universidad Católica de la Santísima Concepción, Concepción, Chile; ^3^Departamento de Ciencias, Universidad Técnica Federico Santa María, Concepción, Chile; ^4^Departamento de Psicología, Universidad de Oviedo, Oviedo, Spain

**Keywords:** predictive model, dropout intention, university students, structural equation modeling, higher education

## Abstract

Dropping out of university studies is one of the current problems of Higher Education; the increased rates during the first year of the study programme is considerable around the world. Dropping out has negative social implications that are reflected at the personal, family, institutional, and educational levels. The aim of this study was to evaluate a predictive model considering the mediation of university social satisfaction and perceived academic performance within the relations between perceived social support, social self-efficacy and academic purposes with career satisfaction and dropout intention in Chilean university students. A non-experimental explanatory design of latent and observed variables was used. Structural equation analyses with Mplus software were performed. The sample consisted of 956 first year university students. The study complied with the ethical requirements for research with human subjects. As a result, a predictive model with adequate adjustment indexes was obtained. When evaluating the explanatory capacity through the coefficient of determination (*R^2^*), it was observed that it explains 38.9 and 27.4% of the variance of the dropout intention and career satisfaction, respectively. This percentage of explanation indicates a large effect size in Social Sciences; therefore, they are considered adequate predictive models. The mediation of university social satisfaction on the relationships between social support, social self-efficacy, and academic purposes with academic adjustment and dropout intention was, respectively, confirmed. The perception of academic performance has less influence on dropout intention and on career satisfaction among first-year students. The model obtained allows explaining the dropout intention and career satisfaction in first year students. In addition, it is composed of variables that can potentially be modified in the interaction of students and professors.

## 1. Introduction

### 1.1. The problem of university dropout in the world

Dropping out of university studies is one of the current problems of Higher Education; the increased rates during the first year of the career is considerable around the world, ranging between 20 and 30% (Micin et al., 2015; Aulck et al., 2017; Arzola, 2019; Bernardo et al., 2020). In Chile, a high incidence of students’ dropout has been found in the first two semesters (39%), then it decreases over time (González-Campos et al., 2020). Dropping out has negative social implications that are reflected at the personal, family, institutional, and educational levels (González-Pérez and Uribe, 2002; Sarcletti and Müller, 2011; Oreopoulos and Petronijevic, 2013), which jeopardize the development of countries. At the family and personal levels, it causes financial and emotional issues that are difficult to overcome in the short term (Cáceres et al., 2019). At the institutional level, it affects the prestige and reputation of the establishments (Angulo-Ruiz and Pergelova, 2013), complicates the educational quality assurance process that is evaluated in Chilean universities, and determine the allocation of public resources (Toledo and Rojas-Palma, 2019).

Dropout intention refers to thoughts, wishes and intentions experienced by students at university concerning the possibility of withdrawing from their degree program before they graduate, or of departing from an institution of higher learning (Mashburn, 2000; Díaz-Mujica et al., 2018). Dropout intention is also understood as part of a decision-making process that unfolds in the early stages of the university experience and that is characteristically dynamic and convergent with multiple factors (Stinebrickner and Stinebrickner, 2014). Dropout intention functions as an indicator of the disposition that a student has as an antecedent of a behavior; and it is based on the attitude toward the behavior, the subjective norm, and the perceived behavioral control (Morales and Correa, 2017). Thoughts associated with dropout can facilitate this disengagement process (Bean and Metzner, 1985).

A central factor of this process is the educator’s role. From Social Cognitive Learning Theory (Bandura, 1986), the reciprocal determinism proposed by Albert Bandura possible to explain how students function through the triadic causal structure: person, environment, behavior. According to the triadic casual structure, students are influenced by the context, which in turn, influences the person and behavior, and at the same time, the student can influence both the context and behavior (Bandura, 2012). Therefore, the interactions, the influences, and the experiences in the university community provide students with models, ideas and sequences of events that can generate an action sequence of learned behaviors for them to feel competent to carry out academic activities and succeed in different demands of the context.

The identification of factors related to university dropout generated the development of theoretical models, such as that of Tinto (1975, 1982), in order to explain this problem. This research is supported by Tinto’s (1987) theoretical model considering that the variables included allow explaining the academic and social integration of students in the first year of Higher Education. Likewise, the variables allow institutions to understand how students’ perceptions shape decisions to persist and how their actions influence those perceptions (Tinto, 2017). Although most theoretical models refer to the university dropout, this study focuses on the investigation of the intention to drop out since it facilitates the understanding of the phenomenon from a preventive approach, i.e., acting before the student dropout. Based on these theoretical models, research with varying degrees of empirical support has been carried out on university students.

### 1.2. Research topic gap

Most research associated with the dropout of university studies have focused on sociodemographic and contextual variables. There is less research on the cognitive-motivational variables of students who can be influenced by teachers in the course of the teaching-learning process (Díaz-Mujica et al., 2019).

A “quick review” was carried out in main or referential databases of interdisciplinary nature (Wos, Scopus, Elsevier) to identify empirical research that have proposed predictive or explanatory models of dropout intention and/or dropout in first year students; it showed that most of the research presented descriptive-correlational and predictive scopes (López-Angulo, 2021). The studies identified have proposed models to explain dropout; however, they present the following limitations: (1) they include variables that are difficult or not possible to modify; (2) the evidence of the models are associative in nature; that is, they conclude that there are significant relationships between variables, but do not explain or clarify how that system of relationships occurs; (3) most of them perform regression analysis, neural networks, machine learning and data mining, based on information from databases, institutional records and retrospective data; and (4) one of their main purposes is to define a successful student’s profile about to be graduated, rather than to identify processes that can be influenced. The review of the literature shows a gap in terms of cognitive and motivational variables that can be modified in the interaction between students and teachers. The scarce research identified (Fisher, 2014; Respondek et al., 2017; Bäulke et al., 2018; Jeno et al., 2018; Díaz-Mujica et al., 2019; Bumbacco and Scharfe, 2020; Fourie, 2020) include different explanatory models of dropout, which are recognized as complex processes determined by individual, institutional and social factors. Notwithstanding, even though the predominant variables are relevant to explain dropout intention, most of them cannot be modified in the interaction of the environment with students; that is to say, they are difficult to change through the teaching-learning process. Only two investigations (Díaz-Mujica et al., 2019; Fourie, 2020) make explicit reference to the quality of some of the variables considered as “modifiable.”

Summarizing the aforementioned, this research is based on the following foundations:

1. the existence of a gap regarding predictive models designed to work with modifiable variables in student-teacher interactions in Latin American, specifically in Chile;
2. the need to contribute to scientific knowledge that substantiate teaching methods in order to foster cognitive-motivational variables and learning;
3. the importance of teachers as models since their behavior and verbalizations facilitate successful academic performance while interacting with students;

Thus, it is relevant to corroborate an empirical model including modifiable variables that have not been analyzed conjointly (e.g., perceived social support, social self-efficacy, academic purposes, social adjustment, academic performance, and academic adjustment). This model will allow an approximation to explain the dropout intention of first-year university students.

### 1.3. Theoretical perspective, background and prior research

Social support is defined as the perception of being assisted by others and having a trusted network whenever one needs support in daily life situations or in moments of crisis (Taylor, 2011). More specifically, social support can come from three main sources: family, friends and significant others (Zimet et al., 1988). Perceived social support predicts and explains academic performance (Richardson et al., 2012; Vander Zanden et al., 2018), as well as the transition during the first year of university (Rodríguez et al., 2017). It is also related to social (Rahat and İlhan, 2016) and career satisfaction (*r* = 0.56, *p* < 0.05; Akanni and Oduaran, 2018).

Career satisfaction involves the degree to which students adapt to academic demands, calibrate their efforts, make a commitment to their studies, and manage their behavior in class (Baker and Siryk, 1989; Credé and Niehorster, 2012). Career satisfaction is positively related to academic performance (Rienties et al., 2012; Bailey and Phillips, 2016; Hazan and Miller, 2017; Páramo et al., 2017; Van Rooij et al., 2018) and academic success (*r* = 0.186, *p* < 0.001; Raza et al., 2020). A relevant sub-variable of career satisfaction is satisfaction with the study program, which has shown a significant and negative relationship to dropout intention (Duque, 2014). Study program satisfaction is related to persistence in one’s university studies (*r* = 0.51, *p* < 0.05; Lent et al., 2016), and career satisfaction is influenced by interaction with teachers and classmates, with Higher Education social satisfaction being a significant determinant of academic performance (Delaney, 2008; Sevinc and Gizir, 2014). The degree to which students integrate into the university’s social structures, participate in campus activities, meet new people and make friends, indicates the level of social satisfaction at university that they are attaining (Baker and Siryk, 1989; Páramo et al., 2017). The students’ relationship with the university environment is significant and the sources of support (i.e., family, friends and significant others) are important for social fit (*r* = 0.16–0.27). The abovementioned social satisfaction impacts positively on career satisfaction (*r* = 0.619, *p* < 0.01).

A variable that influences Higher Education social satisfaction is the student’s social self-efficacy because it enables them to initiate and maintain interpersonal relations with their classmates and other persons of interest (Gecas, 1989; Smith and Betz, 2000). It is conceptualized as the beliefs, perceptions or expectations held by individuals regarding their capacity to organize actions and efforts that are required to materialize a specific type of achievement (Bandura, 1997). This implies having the skill, self-assurance and perceived capacity for grasping and predicting social situations, expressed through behavior that is demonstrative of the individual’s adaptation to social situations (Grieve et al., 2014). Social self-efficacy has been shown to have a positive relation to academic performance (Grieve et al., 2014; Dunbar et al., 2018). A higher degree of self-efficacy leads to being better adjusted to campus life and greater satisfaction with one’s friendships. Social self-efficacy is associated with positive experiences of Higher Education social satisfaction (Meng et al., 2015). Consequently, social self-efficacy is a facilitator of social satisfaction for students admitted to university (Wei et al., 2005; Matsushima, 2016).

Another variable that is pertinent to fitting in and permanence at university are students’ academic purposes. These refer to supra-ordinary, valuable and transcendent aims that promote intentional behaviors that can be structured as specific goals. They enable the student to persevere when faced by obstacles and adversities in the university context, and their self-regulating character thus aids the student in deciding the next course of action they should pursue, what to focus their attention on, and valuing the present but always with a future-oriented horizon (López-Angulo, 2021). Having an intent helps to keep motivation high and to persevere in adverse situations (McKnight and Kashdan, 2009). It impacts positively on students’ perceptions of their academic workload, their participation in academic activities, and on their relationships with teachers and peers (Xerri et al., 2017).

Finally, academic performance is a multidimensional variable that can be influenced by personal factors (i.e., sociodemographic and psychological) and contextual ones (i.e., economic, familial and academic; Mayora-Pernía and Fernández-Morgado, 2015). More specifically, in the first year or semester, academic performance has a high positive correlation with permanence at university (Araque et al., 2009; Bernardo et al., 2016), and is considered an indicator of adaptation to the demands of academic life (Aranda et al., 2013) and one of the best predictors of retention in second and third year (Westrick et al., 2015).

Students who feel socially supported may adapt better to Higher Education (Rodríguez et al., 2017; De Oliveira-Nunes et al., 2020; Lee et al., 2020; Mostert & Pienaar, 2020), and may achieve higher academic performance (Richardson et al., 2012; Abdullah et al., 2014; González-Chong, 2017; Alipio, 2020), which has a positive impact on career satisfaction and decreased dropout intention (Esteban-García et al., 2016; Motl et al., 2018). Similarly, students with high social self-efficacy have beliefs, perceptions and expectations about their abilities to establish social contact easily and develop interpersonal relationships, which is relevant for university social adaptation (Castellanos-Páez et al., 2017; Gazo et al., 2020), and the perception of academic performance (Casas and Blanco, 2016) which at the same time have positive impact on dropout intention. Students with established academic purposes give meaning and direction to academic life, therefore contribute to maintain motivation, and persist in the face of adverse situations (Steger et al., 2006; Hill et al., 2010); possessing academic purposes has a positive impact, as they help and guarantee permanence in studies (Kennett et al., 2013; Xerri et al., 2017).

The empirical evidence available on the relations between perceived social support, social self-efficacy, social adjustment and academic performance make it possible to propose the formulation of a predictive model of career satisfaction and dropout intention. With respect to academic purposes, in view of the paucity of empirical evidence in the academic context, it is important to describe their nature in this specific context, rather than generally, for which an abundance of empirical evidence already exists (DeWitz et al., 2009; Folgueiras and Palou, 2018; García-Alandete et al., 2018; Sun, 2018). This research therefore sought to evaluate model that would consider the mediation of social satisfaction and the perception of academic performance in the relationship of perceived social support, social self-efficacy and academic purposes to career satisfaction and dropout intention among university students. The following hypotheses were established:

*H1.* There is an indirect relationship of perceived social support with career satisfaction and intention to drop out through social satisfaction and perceived academic performance.

*H2.* There is an indirect relationship of social self-efficacy with career satisfaction and intention to drop out through social satisfaction and perceived academic performance.

*H3.* There is an indirect relationship of academic purpose with career satisfaction and intention to drop out through social satisfaction and perceived academic performance.

**Figure 1:**
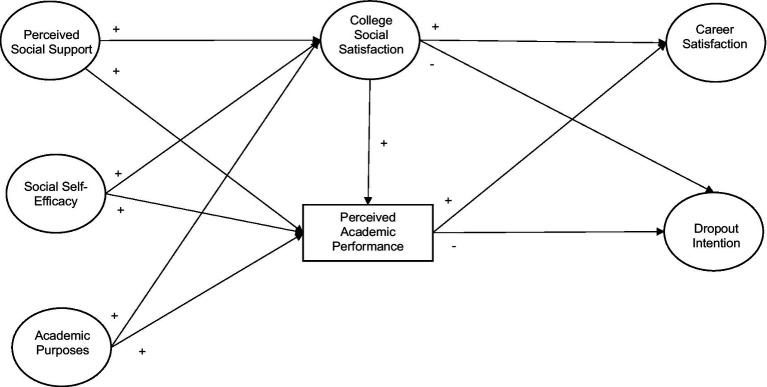
Hypothetical predictive model of dropout intention.

*H4.* Social satisfaction and perceived academic performance mediate the relationship between perceived social support, social self-efficacy and academic purposes with career satisfaction and intention dropout of university students, constituting a predictive model of the relationships between these variables, as shown in Figure 1.

## 2. Materials and methods

The study employed a cross-sectional predictive empirical design with latent variables having a structural model of relationships between variables, and a measurement model that includes the various indicators that define a construct or latent variable. These two models are represented through a system of structural equations, in which some variables are latent and others are observable (Ato et al., 2013).

### 2.1. Participants

The participants were university students of the 2019 cohort attending first semester. The sampling strategy was non-probability. A total number of 1,028 students volunteered to take part in the study. However, 72 were removed as they failed to meet one or more of the following inclusion criteria: (1) first time university students, and (2) outliers, detected using Mahalanobis distance, as it allows the identification of multivariate outliers. The sample consisted of 956 first year university students, see Table 1. Four Chilean universities took part, one state university and three private ones. The total sampling of students consisted of 500 males (52.3%), 454 females (47.5%) and 2 (0.2%) reporting another gender identity. Their ages ranged from 17 years to 23 years (*M* = 18.781; DE = 1.192).

**Table 1:** Description of participants according to area of knowledge, sex, and age.

OECD* Area	*n*	Men	Women	Other’s	Age	SD
Health and medical sciences	129	61	68		18.63	1.02
Natural sciences	52	27	25		19.37	1.03
Social sciences	432	162	268	2	19.03	1.29
Engineering and technology	343	250	93		18.6	1.09
Total	956	500	454	2	18.78	1.19

*Organization for economic co-operation and development.

### 2.2. Instruments

#### 2.2.1. Multidimensional scale of perceived social support

The study employed the version of the Multidimensional Scale of Perceived Social Support (Zimet et al., 1988) validated for Chilean universities (López-Angulo et al., 2021). It is a self-reporting instrument with a Likert-type response scale ranging from 1 = strongly disagree to 7 = strongly agree. The scale has a factorial structure of three second-order factors and a general factor. It contains 12 items that measure the support provided by social relationships established by the individual in his or her environment, the three main sources being family, friends and significant others (Zimet et al., 1988). Some examples of items are: “My family gives me the help and emotional support I require”; “I can talk about my problems with my friends”; “When I need help, I know there is someone who can give me support.” The scale has adequate psychometric properties: *α* = 0.903 (Family), *α* = 0.928 (Friends), and *α* = 0.864 (Significant Others). The score is based on averaging the answers to the 12 items that make up the scale, where the highest scores indicate a greater perception of received social support. The analysis of the measurement model showed good fit indices for a three factor structure [*χ*^2^ = 207.430, *p* < 0.001; RMSEA = 0.055 (90% IC: 0.047–0.063; CFI = 0.957; TLI = 0.945; RSMR = 0.030)] as well as reliability *ω* = 0.891 (Family), *ω* = 0.923 (Friends), and *ω* = 0.851 (Significant Others).

#### 2.2.2. Cognitive and behavioral social self-efficacy questionnaire

The Cognitive and Behavioral Social Self-Efficacy Scale (Grieve et al., 2014) was employed that is validated for Chilean universities (López-Angulo et al., 2021). It measures students’ beliefs, perceptions and expectations regarding their skill sets for easily establishing social contact and developing interpersonal relationships in the university context (Gecas, 1989; Smith and Betz, 2000), as well as the perceived ability, self-confidence and capacity for grasping and predicting social situations, expressed through behavior (Grieve et al., 2014). The inventory comprises 18 items. In each item the respondent is asked to give a numeric response as to how sure they are that they can achieve what is described in each item, with “1” meaning “Not at all sure” and 5 meaning “Very sure.” The instrument for measuring social self-efficacy is configured by three interrelated dimensions, which in turn configure a global construct. A second-order factorial model was confirmed, consisting of 15 items, in which factor 1 measures aspects related to the capacity to predict others’ behavior, factor 2 refers to cognitive elements related to the perceived capacity to understand others’ feelings, and factor 3 captures behaviors related to perceived ability and self-confidence in social situations, and which are expressed through the individual’s behavior and are indicative of fitting into social situations. A sample item: “In interactions with others in the study program, I can: ‘Anticipate the behavior of other persons’, ‘Understand the feelings of other persons’, and ‘Easily adapt to social situations’.” The fit indices of the academic commitment scale were: *χ*^2^ (83) = 452.500, *p* < 0.001; CFI = 0.963; TLI = 0.954; RMSEA = 0.066 (0.060–0.072), SRMR = 0.035. This scale has adequate psychometric properties: *α* = 0.811 (Prediction), *α* = 0.816 (Cognitive), *α* = 0.824 (Behavioral), and *α* = 0.870 (General). The score is based on the average of the answers, with the highest scores indicating more social self-efficacy. The analysis of the measurement model showed good fit indices for a three factor structure [*χ*^2^ = 452.500, *p* < 0.001; RMSEA = 0.066 (90% IC: 0.060–0.072; CFI = 0.963; TLI = 0.954; RSMR = 0.035)] as well as reliability *ω* = 0.812 (Factor 1), *ω* = 0.816 (Factor 2), and *ω* = 0.828 (Factor 3).

#### 2.2.3. Student adaptation to college questionnaire

To measure university social satisfaction, career satisfaction and academic purposes, subscales validated in Chilean universities (López-Angulo, 2021; López-Angulo et al., 2021) of the Student Adaptation to College Questionnaire (SACQ; Rodríguez et al., 2012) were used. The university social satisfaction scale is comprised of 7 items configuring a first-order model that enable measuring student satisfaction with social activities at university. Sample items are: “I think I fit in well in my university”; “I’m content with my participation in the social activities offered by the university” *α* = 0.806. Satisfaction with the study program was measured with a subscale of the dimension of academic fit. This scale is comprised of 4 items: “I’m satisfied with the number and variety of my subjects”; “I’m very satisfied with the teachers that I have this year.” Academic purposes were measured with a subscale of the dimension of academic fit. This scale is made up of 4 items. Sample items: “My academic objectives and intentions are well defined.” “I know why I’m in college and what I want to get out of it.” *α* = 0.798. For all the subscales, a response scale of 7 alternatives was used (from 1 = Totally disagree to 7 = Totally agree). The score was obtained through the average of the responses to each factor. A higher score indicates that the student has better-defined academic purposes. The analysis of the measurement model showed good fit indices for all factors. For factor structure social satisfaction [*χ*^2^ = 43.396, *p* < 0.001; RMSEA = 0.047 (90% IC: 0.031–0.063; CFI = 0.977; TLI = 0.996; RSMR = 0.026)] and reliability (*ω* = 0.814). Also, for the career satisfaction [*χ*^2^ = 103.239, *p* < 0.001; RMSEA = 0.047 (90% IC: 0.037–0.058; CFI = 0.964; TLI = 0.951; RSMR = 0.042)], as well as reliability (*ω* = 0.824); and the academic purposes [*χ*^2^ = 2.282, *p* < 0.001; RMSEA = 0.012 (90% IC: 0.000–0.067; CFI = 0.000; TLI = 0.999; RSMR = 0.009)], as well as reliability (*ω* = 0.814).

#### 2.2.4. Perception of academic performance

The perception of academic performance was obtained through the grade reported by the students, which responded to their self-evaluation of their performance during the current semester. The item read: Mark with an x the box that best fits your academic performance. The Chilean grading system was used, of a continuous variable that goes from 1 = Very deficient, to 7 = Excellent. Academic performance was classified as low for grades lower than 3.0, average in the range of 3.1 to 5.9, and high between 6.0 and 7.0.

#### 2.2.5. Dropout intention

Dropout intention was measured using 3 *ad hoc* items that sought to detect whether a student had any intention or wish to discontinue his or her studies (Díaz-Mujica et al., 2019). They were the following: “I’m thinking of leaving the program,” “I’m thinking of applying to the same program in a different university,” “I’m thinking of attending another university and applying to a different degree program.” A response scale of 7 alternatives was used (from 1 = Totally disagree to 7 = Totally agree). The score was calculated based on the average of the responses to the 3 items. The correlations were statistically significant between items 1 and 2 (*r* = 0.420; *p* < 0.001), 1 and 3 (*r* = 0.545; *p* < 0.001) and items 2 and 3 (*r* = 0.544; *p* < 0.001) as well as the reliability (*ω* = 0.765).

### 2.3. Procedure

In order to access the sample, the authorities of the different faculties were contacted to obtain the appropriate permissions and carry out the study. The selection of participants was based on convenience and accidental sampling. The survey takers coordinated with the teachers and a space was designated that students were invited to go to in order to take part in the study. The students’ informed consent was obtained beforehand that considered the ethical principles established by the Singapore Declaration and the National Research and Development Agency of Chile. Reference was also made to data protection and the use of the data solely for research purposes. Following this, the students who volunteered to participate answered the questionnaires in the classrooms with pencil and paper. The application of the questionnaires was carried out in the first semester.

### 2.4. Analysis plan

The study’s objective was to evaluate a predictive model that would consider the mediation of social satisfaction and the perception of academic performance (mediating variables) in the relationship of perceived social support, social self-efficacy and academic purposes (predictor variables) to career satisfaction and dropout intention among university students (criterion variables).The research objectives were tested by means of structural equation modeling (SEM), which, as a multivariate statistical method, combines factor analysis and multiple regression to simultaneously examine relations of interdependence between the observed and latent variables, as well as between the latent variables (Hair et al., 2014). The structural model was evaluated after the measurement model was accepted. Firstly, analysis was carried out of the measurement model, second, the analysis of the structural model, and third, the mediation analysis through the Sobel test.

For the SEM, the five steps recommended in the literature for the analysis of structural equations were applied: specification, identification, estimation, evaluation and modification of the model (Bollen and Long, 1993; Kline, 2015). Specification consisted of formulating an initial hypothetic model based on the theory and empirical findings reviewed. Identification concerned the examination of whether there was enough information to enable contrasting the model. When calculation of the degrees of freedom was done, it was observed that these were greater than zero (df = 921), exactly as the literature suggests. An “over-identified” model was thus observed, whose fit can be submitted statistically to verification. Estimation was done with the ML estimator (Maximum Likelihood Estimation), considering that the variables were ordinal, measured on a Likert scale of up to 7 points, and, thus, treated as continuous measures (Sass et al., 2014). Specifically, the MLR (Maximum Likelihood Estimation Robust) was used, given the robustness it offers with a multivariate data distribution that is not so similar to a normal distribution, and given the possibility of visualizing the standard errors.

An evaluation was done of the factorial structure underlying a matrix of correlations for each latent variable through the CFA. Based on the literature, the fit indices of the proposed model are useful for determining an optimal model: (1) Non-significant Chi-values $\left(X^{2}\right)$ *p* ≥ 0.05 (Tabachnik and Fidell, 2007), (2) root mean square error of approximation (RMSEA) values less than 0.07 (Hu and Bentler, 1999; Steiger, 2007), (3) comparative fit index (CFI) and non-normalized fit index (TLI) should be greater than 0.94 (Hair et al., 2014), and (4) item factor loadings should be significant equal to or greater than 0.30 (Field, 2013), preferably greater than 0.40 (Hair et al., 2014; Lloret-Segura et al., 2014).

Mediation effects were verified with the Sobel Test, which uses the multivariate delta method to calculate the standard error of the indirect effect (Sobel, 1982). The bootstrap method is preferred over other as it does not impose the assumption of normality of the sampling distribution of indirect effects, has a lower type I error rate, and has greater power to detect mediation (MacKinnon et al., 2002). Total indirect effect is the mediation effect of the set of mediators. Specific indirect effect is the unique mediator effect of a mediator above and beyond other mediators in the model. Bias-corrected bootstrapped 95% confidence intervals of the indirect effects were derived from 5,000 resamples. If the interval does not include zero, a mediated effect is considered significant. Mplus software version 8.4 (Muthén and Muthén, 1998/2017) was used for all analyses.

## 3. Results

### 3.1. Preliminary analyses: Descriptive statistics

The students’ scores for most of the variables were moderate: they answered that they slightly agreed with regard to the social support they received from family, friends and significant others; they were slightly satisfied, they fit in academically and socially to the demands of the university; and their academic purposes were moderately defined. They perceived their level of academic achievement as moderate/sufficient, and as for social self-efficacy in relationships with others, they felt undecided and unsure about socially interacting with other students in the study program. Finally, the students reported low intentions of dropping out, see Table 2.

**Table 2:** Descriptive statistics of the scores of the variables.

Variables	Min	Max	*M*	*SD*
Perceived social support	1.08	7.00	5.600	1.131
Academic purposes	1.67	7.00	5.774	0.9132
Social self-efficacy	1.00	5.00	3.620	0.5933
Social satisfaction	1.43	7.00	5.013	0.9787
Perceived academic performance	1.00	7.00	4.800	1.09
Career satisfaction	1.00	7.00	5.339	1.097
Dropout intention	1.00	7.00	1.681	1.141

Considering the scores in the “high” category (≥ 6), “median” (≥ 3.1 and ≤ 5.9) and “low” (≤3), it was observed that 44.7% reported having high perceived levels of social support, 27.2% had high levels of social self-efficacy, 22.1% had high perceptions of academic performance, 54.7% stated they had well-defined academic purposes, 15% high social satisfaction at university, 35.3% were highly satisfied with the degree program, and 4.9% evaluated their academic performance as of a high level. While these scores do not point to many the students rating their levels as “low,” there is definitely a significant percentage of students with median scores. For example: career satisfaction 59.8%, evaluation of academic performance 78.8%, social satisfaction at university 77.7%, academic purposes 43.1%, social support 49.4%, social self-efficacy 68.6%, and perception of academic performance 54.5%.

As shown in Table 3, the results indicate the presence of correlations (i.e., statistically significant relationships) between the predictor and dependent variables. Such relationships are an essential prerequisite for verifying a predictive model. The items with the highest relation to dropout intention were academic purposes (*r* = −0.338) and university social satisfaction (*r* = −0.314). Evaluation of academic performance presented significant correlations with the perception of academic performance (*r* = 0.542) and university social satisfaction (*r* = 0.371). Satisfaction with the study program presented a medium correlation with academic purposes (*r* = 0.318) and university social satisfaction (*r* = 0.360).

**Table 3:** Correlations between model variables.

Variables	1	2	3	4	5	6	7
Perceived social support	1						
Social self-efficacy	0.258^**^	1					
Academic purposes	0.270^**^	0.264^**^	1				
Social satisfaction	0.419^**^	0.491^**^	0.429^**^	1			
Perceived academic performance	0.261^**^	0.152^**^	0.242^**^	0.363^**^	1		
Career satisfaction	0.211^**^	0.184^**^	0.318^**^	0.360^**^	0.250^**^	1	
Dropout intention	−0.163^**^	−0.095^**^	−0.338^**^	−0.314^**^	−0.247^**^	−0.261^**^	1

^**^Correlation is significant at the 0.01 level (bilateral).

Regarding the degree of correlation between the predictor variables, no extremely high relationship is observed (i.e., more than 0.80 or 0.90) between the variables of social support, social self-efficacy and academic purposes, that would indicate multicollinearity (Field, 2013).

### 3.2. Predictive model of dropout intention and career satisfaction: Structural equation modeling

The study’s objective was to evaluate a predictive model that would consider the mediation of social satisfaction and the perception of academic performance in the relationship of perceived social support, social self-efficacy and academic purposes to career satisfaction and dropout intention among university students.

The model evaluation involved analyzing the results of the estimated fit, based on the absolute and incremental fit indices obtained. As can be seen, the chi-squared is significant and contrary to expectations. However, its sensitivity to erroneous specifications in large models is well known (Saris et al., 2009). Therefore, the comparative indicators or the lack of fit were evaluated to verify the model’s results. The RMSEA ≤0.07 (Hu and Bentler, 1999; Steiger, 2007) is adequate, likewise the SRMR <0.08 (Hair et al., 2014). The CFI and TLI indices did not show optimal adjustments, since their values ought to be ≥0.94 (Hair et al., 2014).

The 20.5 and 22.5% variance in dropout intention and career satisfaction, respectively, were explained upon evaluating the model’s explicative capacity through the determination coefficient (*R*^2^). The percentage explanation indicates a low effect size; therefore, the modification or re-specification of the structural model was proposed, with the aim of adding or eliminating parameters that would facilitate obtaining a parsimonious model, one that would explain, to a greater extent, career satisfaction and dropout intention.

**Figure 2:**
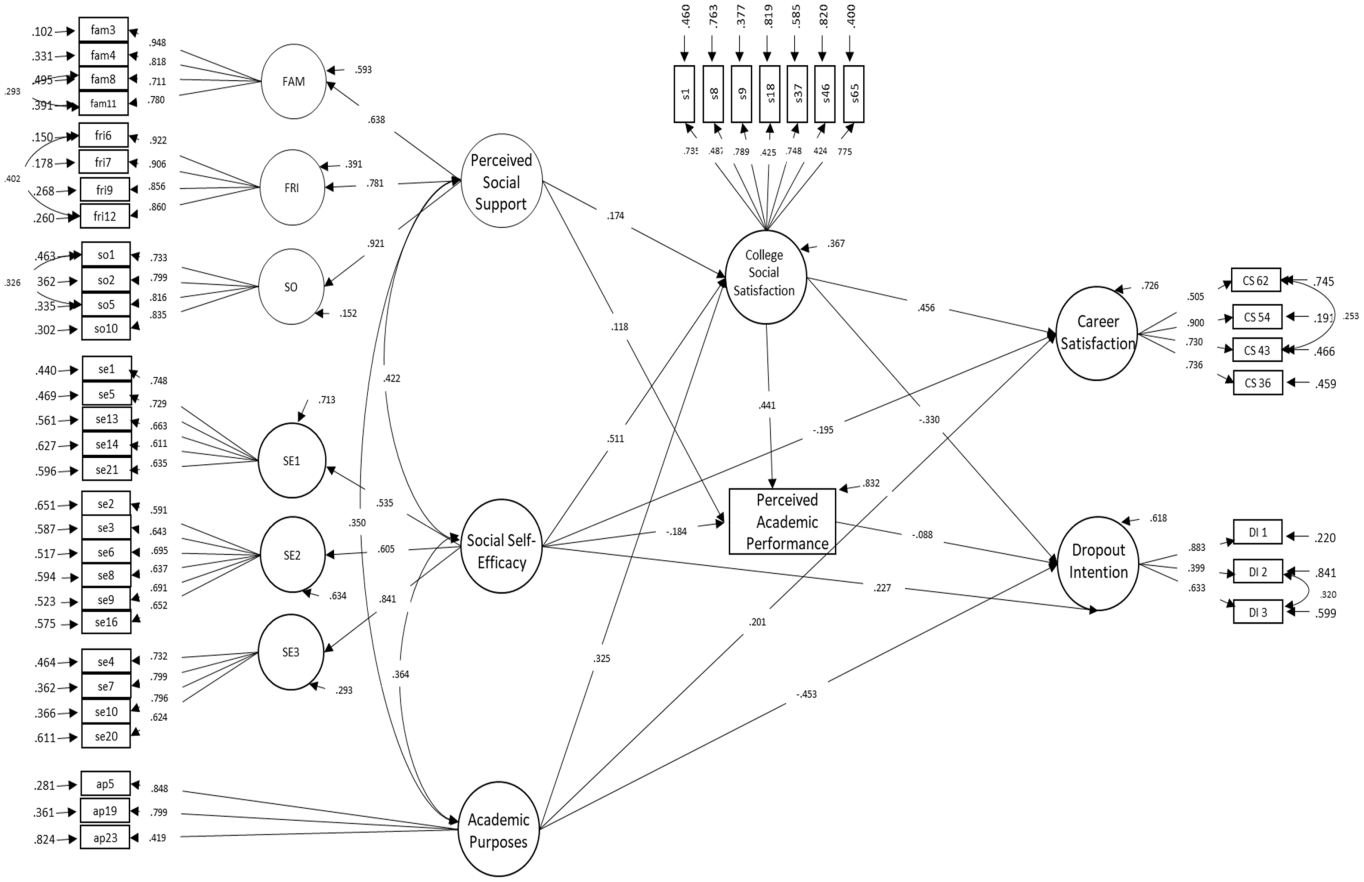
Predictive model of the dropout intention of Chilean university students.

For model re-specification, the routes indicated by Mplus were considered through the analysis of the modification indices, and relations were added only considering their theoretical meaning. In this way, direct relationships were added among some of the predictor and the dependent variables. This was done, on the one hand, with the dropout intention, specifying the routes: social self-efficacy → dropout intention; academic purposes → dropout intention. On the other hand, career satisfaction was specified with the routes: social self-efficacy → university social satisfaction, and academic purposes → career satisfaction. In addition, the following residuals were correlated: DI2 and DI3 (IM = 50.698), FAM8 and FAM11 (IM = 49.551), SO1 and SO2 (IM = 37.487), y CS43 and CS62 (IM = 39.441). The wording of these pairs of items alludes to dropout intention, support from the family, from significant others, from friends, and career satisfaction, respectively. The correlations of residuals were included based on the inspection of modification indices. A model with adequate fit indices was thus obtained (Table 4, graphical representation in Figure 2), as well as significant relationships, standardized regression weights (β), variances and factor loadings.

**Table 4:** Fit indices of the predictive model of career satisfaction and dropout intention.

Models	X^2^	Df	RMSEA	90% CI	SRMR	CFI	TLI
Hypothesized model	2484.574**	926	0.042	0.040–0.044	0.061	0.903	0.896
Final model	2099.779**	914	0.037	0.035–0.039	0.055	0.926	0.920

Df, degrees of freedom of the model; RMSEA, root mean square error of approximation; (90% CI), 90% confidence interval for RMSEA; SRMR, standardized root mean square error of approximation; CFI, comparative fit index; TLI, Tucker-Lewis index; **p < 0.001.

As can be observed in Figure 2, social support relates positively to university social satisfaction (*β* = 0.174; *p* ≤ 0.001), which, in turn, relates positively to career satisfaction (*β* = 0.456; *p* ≤ 0.001), but relates negatively to dropout intention (*β* = −0.330; *p* ≤ 0.001). In addition, social support is positively related to perceived academic performance (*β* = 0.118; *p* ≤ 0.05), which, in turn, is negatively related to dropout intention (*β* = −0.088; *p* ≤ 0.05). Social self-efficacy has a positive relationship to university social satisfaction (*β* = 0.511; *p* ≤ 0.001) and dropout intention (*β* = 0.227; *p* ≤ 0.01), and relates negatively to career satisfaction (*β* = −0.195; *p* ≤ 0.05) and with perceived academic performance (*β* = −0.184; *p* ≤ 0.05). Academic purposes relate positively to university social satisfaction (*β* = 0.325; *p* ≤ 0.001), career satisfaction (*β* = 0.201; *p* ≤ 0.001) and relate negatively to dropout intention (*β* = −0.453; *p* ≤ 0.001). In addition, no significant relationship was found between career satisfaction and dropout intention (*β* = −0.014; *p* = 0.775), between perception of academic performance and career satisfaction (*β* = 0.070; *p* = 0.097), neither between academic purposes and perceived academic performance (*β* = 0.013; *p* = 0.814).

After evaluating the explicative capacity of the model presented through the determination coefficient (*R*^2^), it was observed that the model specified explained 38.9 and 27.4% of the total variance of dropout intention and program satisfaction, respectively.

### 3.3. Analysis of direct and indirect effects: Mediation analysis

As observed in Table 5, the total indirect effects of social self-efficacy, and academic purposes on dropout intention were significant in the Sobel Test and the 95% bootstrapping did not contain zero. This result, coupled with a significant direct link, indicated that social satisfaction mediated the social self-efficacy and academic purposes whit dropout intention relationships.

**Table 5:** Confidence intervals of standardized total, total indirect, specific indirect, and direct effects for the final model.

Model routes	Effects total	Effects total indirect	Effects direct	Effects indirect specific
Perceived Social Support → Social Satisfaction → Dropout Intention	−0.022 [−0.108, 0.062]	−0.076 [−0.118, −0.044]	0.054 [−0.032, 0.137]	–
Social Self-efficacy → Social Satisfaction → Dropout Intention	0.054 [−0.040, 0.142]	−0.173 [−0.312, −0.083]	0.227* [0.101, 0.387]	−0.168 [−0.309, −0.077]
Academic Purposes→ Social Satisfaction→ Dropout Intention	−0.579* [−0.666, −0.486]	−0.126 [−0.195, −0.077]	−0.453* [−0.560, −0.343]	−0.107 [−0.187, −0.058]
Social Satisfaction→ Perceived Academic Performance → Dropout Intention	−0.375* [−0.557, −0.235]	−0.046 [−0.096, 0.001]	−0.330* [−0.535, −0.176]	–
Perceived Academic Performance→ Dropout Intention	−0.089 [−0.159, −0.014]	−0.001 [−0.010, −0.004]	−0.088 [−0.160, −0.012]	–
Career Satisfaction → Dropout Intention	−0.014 [−0.089, −0.075]	0.000 [0.000, 0.000]	−0.014 [−0.089, 0.075]	–
Perceived Social Support → Social Satisfaction → Career Satisfaction	0.104* [0.028, 0.178]	0.093 [0.057, 0.137]	0.011 [−0.069, 0.092]	–
Social Self-efficacy → Social Satisfaction→ Career Satisfaction	0.041 [−0.046, 0.130]	0.236* [0.128, 0.381]	−0.195* [0.363, −0.038]	0.233* [0.126, 0.379]
Academic Purposes → Social Satisfaction → Career Satisfaction	0.360* [0.273, 0.442]	0.159 [−0.110, 0.225]	0.201* [0.105, 0.292]	0.148* [0.098, 0.219]
Social Satisfaction→ Perceived Academic Performance → Career Satisfaction	0.487* [0.339, 0.663]	0.031 [0.003, 0.065]	0.456* [0.299, 0.642]	–
Perceived Academic Performance→Career Satisfaction	0.070 [−0.003, 0.141]	0.000 [0.000, 0.000]	0.070 [−0.003, 0.141]	–

CI, confidence interval. *The 95% CI does not go through 0.

The indirect link of social self-efficacy, and academic purposes on career satisfaction were significant in the Sobel Test, and the 95% bootstrapping did not contain zero. This result, coupled with a significant direct link, indicated that social satisfaction mediated the social self-efficacy and academic purposes whit career satisfaction relationships.

## 4. Discussion

This section presents the analysis of the main findings yielded by the study’s proposed objectives; the study’s limitations and implications; future lines of research and conclusions.

### 4.1. Perceived social support, university social satisfaction, perception of academic performance with career satisfaction and dropout intention

The study’s results did not show statistically significant indirect effects of social support on career satisfaction and on dropout intention through university social satisfaction nor perceived academic performance; therefore, hypothesis

*H1:* There is an indirect relationship of perceived social support with career satisfaction and intention to drop out through social satisfaction perceived academic performance, was not confirmed.

Even if it is true that there are several variables that interact to predict dropout intention, many studies have observed direct effects between the different variables, but not the mediating effects between them and dropout intention (Bernardo et al., 2022). The model results showed that social support relates positively to university social satisfaction (β = 0.174; p ≤ 0.001) and relates negatively to dropout intention (*β* = −0.330; *p* ≤ 0.001). In addition, social support is positively related to perceived academic performance (*β* = 0.118; *p* ≤ 0.05), which, in turn, is negatively related to dropout intention (*β* = −0.088; *p* ≤ 0.05). This result indicates that social support could directly influence on dropout intention thus showing to the importance of support from family, friends and significant others for students in the first years. The finding is consistent with that of prior studies, that perceived social support from family, friends and fitting into university (academically, socially) are predictive of the transition during first year (Rodríguez et al., 2017; De Oliveira-Nunes et al., 2020). The support received from teachers, classmates, and good relationships at university contribute to permanence (Esteban-García et al., 2016; Motl et al., 2018).

The present study confirmed the relationship between perceived social support and the perception of academic performance, as prior research has done (Abdullah et al., 2014; González-Chong, 2017; Rodríguez et al., 2017; Alipio, 2020). The fact that perception of academic performance and social satisfaction had not mediated the relationship between social support and intention of drop out and career satisfaction can be explained by the large number of variables included in the model. This is in contrast with other studies that limit the analysis to pure relations or to a small number of strongly related variables. The above result may also be indicative of a contrary relationship; i.e., that even if the student may be dissatisfied with the program or have low social satisfaction, he/she may have a high perception of academic performance; at least in the first semester of the academic year in which the measurements in this study were taken.

### 4.2. Social self-efficacy, university social satisfaction, perceived academic performance with career satisfaction and dropout intention

The results showed statistically significant indirect effects of social self-efficacy on satisfaction with the career and on dropout intention through satisfaction university social. However, perceived academic performance did not prove to be a mediating variable; therefore, hypothesis

*H2:* There is an indirect relationship of social self-efficacy with career satisfaction and intention to drop out through social satisfaction and perceived academic performance, was partially confirmed.

This finding show up of the importance of beliefs, confidence and the perceived ability to understand and predict social situations that facilitate establishing social contact and developing interpersonal relationships in the university context. Worthy of note is the correlation between social self-efficacy and university social satisfaction. Social Cognitive Theory provides confirmation and can explain the value of social interactions in the immediate context of human development, which in this case is the university as the locus of students’ formative period. Some researches confirm this result (Meng et al., 2015; Matsushima, 2016).

The reciprocal determinism put forth by Albert Bandura explains how students function through the triadic causal structure of individual, environment, behavior. They are influenced by context, which, in turn, influences the individual and his or her behavior, and, at the same time, the student can influence both context and behavior (Bandura, 2012). Therefore, the interactions, influences, experiences in the university community as what can provide students with models, ideas and sequences of events that can generate a chain of learned behaviors, such that they feel and believe themselves competent to carry out academic activities and successfully meet the varied demands of the context.

These results confirm the findings of a study on how personal and cognitive factors mediate the relationship between students and their environment, aside from emphasizing the importance of social interactions for developing human capabilities (Medrano, 2011). However, in this research there was no verification of the mediating effect of perceived academic performance on the relationship of social self-efficacy to career satisfaction and dropout intention.

In this respect, ever since Bandura’s postulates and the empirical testing performed in several studies (Castellanos-Páez et al., 2017), the positive impact of beliefs on personal efficacy cognitive, motivational and academic performance variables has become widely known. The same is true for theoretical studies (Casas and Blanco, 2016) on the link between self-efficacy and academic performance in different areas and at different levels of educational endeavor. It would seem that the type of self-efficacy, or better yet, the specific domain of self-efficacy, is important for understanding the loci of the student’s beliefs, perceptions and assurance of success. In this regard, Bandura (2006) and Bandura (2012) drew attention to the need for instruments measuring self-efficacy to cover specific domains of performance and the context in which behavior is deployed. It may be inferred that, while social self-efficacy is important for socially fitting into the context, the perception of academic performance is not a variable that mediates the relationship between social self-efficacy, dropout intentions and career satisfaction.

It should be pointed out that 68.6% of the study participants obtained average scores in social self-efficacy. With respect to levels of social self-efficacy vis-à-vis educational attainment, statistically significant differences were detected in favor of second year students, which is possibly associated with the latter’s higher motivation to establish social relationships (Gazo et al., 2020). Regarding to this point, Bandura (2012) has indicated that people with high self-efficacy have more flexible strategies for managing their environment: they gather more knowledge, are motivated to achieve goals and perform complex tasks, in contrast to persons with low self-efficacy, who tend to avoid such tasks.

It can be inferred from these results that students who possess high social self-efficacy can more easily initiate and maintain relationships or social contacts, implying a better degree of socialization in the study program and on campus. This can equip them for developing adaptive attributes, such as understanding rules and university culture, enjoying activities and spaces, forming groups to carry out academic activities and the desire to stay at the university.

### 4.3. Academic purposes, perception of academic performance with career satisfaction and dropout intention

The results indicated an indirect relationship of academic purposes with career satisfaction and dropout intention, mediated by university social satisfaction. However, the perception of academic performance did not mediate these relations; therefore, hypothesis

*H3:* There is an indirect relationship of academic purpose with career satisfaction and intention to drop out through social satisfaction and perceived academic performance, was partially confirmed.

Based on these results and considering that 22.1% of the study sample presented high perceptions of academic performance and 54.7% presented well-defined academic purposes, it can be deduced that students who are aware of why they are in university and what they wish to obtain from their studies do not perceive academic performance as being a core aspect for the achievement of their academic purposes. They can imagine themselves carrying out academic activities, consciously defining goals and aspirations, minimizing dropout ideations.

The variable of academic purposes presented a positive direct effect on university social satisfaction. This means that students attribute importance to their fitting in and satisfaction with the university’s social activities, and to having close personal relationships and being actively involved in university life. This correlation seems cogent because it is understood that intentions allow one to define aims, objectives, and develop behaviors that imbue academic life with meaning, and thus enable one to persevere in the face of adversity (Steger et al., 2006). On the other hand, as social beings, students’ adaptation process to university unfolds in the interaction with another (whether an acquaintance, classmate, friend or teacher), and thus it stands to reason that they assign value to social relationships in the university context. These aspects increase the likelihood that students will not show any dropout intentions.

One of the most valuable findings of this research is the moderate correlation between academic purposes and dropout intention (*r* = −0.453; *p* ≤ 0.05). As described in the theoretical section, there is vast empirical evidence from studies of life purpose as mobilizer and developer of personality. However, there have been few such studies carried out on the university domain or context. The present research confirms the positive impact of having academic purposes, as supporting and guaranteeing permanence in university studies. These results coincide with those of Xerri et al. (2017) that a sense of purpose is a motivating factor of student participation in academic activities and social relationships, and thus constitute confirmation of the importance of effective teacher-student relationships.

### 4.4. A model of university career satisfaction and dropout intention in university students

This study’s findings showed to a large extent an adequate fit to the data, and most of the foreseen trajectories were significant. An explicative model of career satisfaction and dropout intention was designed and applied with high percentages of explanation in Social Sciences (Field, 2013). Said model is optimal for the explanation of career satisfaction and dropout intention in first year university students. Therefore, the hypothesis

*H4:* Social satisfaction and perceived academic performance mediate the relationship between perceived social support, social self-efficacy and academic purposes with career satisfaction and intention dropout of university students, constituting a predictive model of the relationships between these variables, as have showed in Figure 1; was confirmed.

The results confirm and emphasize the importance of aspects of the academic and social system relative to the dropout model of Tinto (1987), such as the interactions with peers and with teachers, and social integration in general, given the importance of the others to cushion the impact of the changes implied by adaptation to university. Tinto (2017) suggests certain central variables through which the educational institution can favor the students’ persistence until completion of their studies. He points out the importance of motivation, personal goals, self-efficacy, of involvement or a sense of belonging (i.e., feeling that one is part of the community: the faculty, professors, students), the perception of social support and the perception of the curriculum. The present study confirms the need to reinforce students’ interactions and bonding with their peers and with their teachers. A study in German university students showed the relevance of social and academic integration for decision making regarding staying or dropping out of university; they found that academic and social integration predict dropout intention, with academic interest in their field of study, and social integration with peers, being the most relevant subdimensions (Piepenburg and Beckmann, 2021).

From the students’ perspective, goals and motivation are important for persevering in their university studies (Tinto, 2017). The meaningfulness of this approach is made apparent upon observation of the significant correlation between academic purposes and dropout intention in this research model. Academic purposes as long-term projects give meaning and direction to the students’ academic life, enabling them to persist despite diverse challenges and adversities and, as well, lead them toward strategically delimiting the goals, objectives and behaviors necessary for materializing said intentions.

The model that has been designed and applied contributes toward explaining university dropout intention in Chile. Given the current lack of explicative, mediation and complex analysis models, it fills a gap and sheds light on the role of certain cognitive and motivational variables that can be modified in teacher-student interactions. This thesis provides information on the Chilean context and complements the results of other studies (Díaz-Mujica et al., 2019; Fourie, 2020). One of the strengths of this research is that it focused on first year students (Credé and Niehorster, 2012).

Regarding amount of explained variance, previous studies (Lozano-Medellín, 2010; Fisher, 2014; Respondek et al., 2017; Bäulke et al., 2018; Jeno et al., 2018; Díaz-Mujica et al., 2019; Bumbacco and Scharfe, 2020; Fourie, 2020; Jimenez-Rodriguez et al., 2021; López-Aguilar and Álvarez-Pérez, 2021; Bernardo et al., 2022; Maluenda-Albornoz et al., 2022), have evidenced significant relationships, and explained percentages of variance averaging 21%. This indicates the need for further research to understand or explain the phenomenon of dropout intention more deeply or with greater variance. In this research, 38.9% was obtained, which is considered high percentage in the area of Social Sciences and represents a higher percentage of explanation compared to that reported in similar models. Among the factors that these previous studies have identified as most relevant for predicting dropout, metacognition and self-regulation (Jimenez-Rodriguez et al., 2021), academic burnout and self-efficacy expectations ( López-Aguilar and Álvarez-Pérez, 2021), burnout, disengagement, and attachment anxiety (Bumbacco and Scharfe, 2020) were found. Most studies address variables related to academic motivation, mental health, sociodemographic, academic engagement, satisfaction of basic needs, and academic achievement. Although scarce, studies exploring motivational cognitive variables were identified: (a) Díaz-Mujica et al. (2019) addressed motivation, performance, self-efficacy, self-regulation, and career satisfaction; the variables with the strongest relationships were career satisfaction (*d* = 0.645) and intrinsic motivation (*d* = 0.249); (b) Bäulke et al. (2018) found that the most relevant variables were academic procrastination (*R*^2^ = 0.16), and motivation regulation (*R*^2^ = −0.38); (c) Bernardo et al. (2022) reported that career satisfaction and expectations (*d* = 0.70) and career engagement (*d* = 0.17) were the most predictive variables of dropout intention; and (d) Nemtcan et al. (2020) found that variables such as academic skills (*β* = −0.074), academic self-efficacy (*β* = −0.434), and students’ integration (*β* = −0.287) were the most relevant.

This study is also pertinent as it addresses the issues of intent, ideation, or the possibility of academic dropout among university students. It was decided that the study should focus on intention due to the link between this phenomenon and the Theory of Reasoned Action (Ajzen and Fisbein, 1980) and its complement, the Theory of Planned Behavior (Ajzen, 1991; Mc Eachan et al., 2011). Emphasis was placed on the fact that intention is a predictor of behavior, since it is an indicator of predisposition. The intention to deploy behavior arises from three elements: the individual’s attitude toward the behavior, the subjective norm, and the perception of control.

Given the need to take measures in order to facilitate fitting into the university and avoiding dropout intention, changes can be implemented *via* three intervention modalities: promotion, prevention and correction. Attention can also be given to covering needs for social, psychological and educational support to ensure students’ success and completion of their first year courses (Gomes-de Barros and Almeida, 2021). There are alternatives that universities can implement to structure first-year students’ campus life. In this regard, innovation and creativity are important and should consider the characteristics of the context (Gómez-de Salazar and Álvarez-Gil, 2020).

The contribution of the study with respect to previous literature is the identification of variables that allow us to delve into the study of dropout intention that confirms the relevance of the social variables over the academic ones, especially in the first semesters higher education. A particularity of these variables consists of the possibility of change them in the classroom through the teaching-learning process and social relationships among peers. From the psychometric perspective, this study provides researchers in the Latin American region with a valid and reliable scale to measure the variables: perceived social support, social self-efficacy, academic and social university satisfaction. At a theoretical level, it proposes a new model that helps to progress in research on the influence of cognitive-motivational and social variables and their influence on dropout. Especially considering that there are multiple studies that address the influence of affective-motivational variables on the intention to drop out, but there are few studies that address the relationship between cognitive variables and their influence on the intention to drop out (Nemtcan et al., 2020).

The study of dropout in Latin American context is relevant, the university dropout rate ranges between 20 and 30% (SIES, 2014, 2019), which is very high; and despite initiatives to mitigate dropout and its negative social causes, they remain to be few Latin America. There are gaps in the quality of education related to the IVE (vulnerability index), with technological advancement and infrastructure that account for an education system with significant precariousness. Inequalities in access to digital resources such as Internet or computers; inequalities among private, elite public, or traditional state offers, as well as gaps between rural and urban areas (Esper et al., 2022).

### 4.5. Limitations and future research proposals

One limitation was that the issue of variable changes over time was not addressed. This was due to the cross-sectional design employed in which data was obtained from a single time period; due to the cross-sectional design, inferences regarding causality should be made with caution. A considered second limitation was the social desirability bias that may have influenced the measurement of perceived academic performance (Imose and Barber, 2015).

Future research could employ longitudinal designs which will enable accounting for the possible trajectories of certain variables vis-à-vis dropout intention and consummate dropout. Other types of designs (i.e., qualitative) will enable in-depth exploration of the relationship between social self-efficacy and academic performance. Explicative models can be tested and estimated considering careers of different knowledge areas. It would likewise be desirable to conduct research on a more heterogeneous sampling, such as considering the types of degree programs in underrepresented universities. Another potential area of future study is the analysis of how sociodemographic and personal variables can function as moderators of existing relationships between the model’s variables, and contrasting the differences between perceived academic performance and semestral academic performance. Other research studies could explore the differences among universities belonging to the CRUCH (Council of Rectors of Chilean Universities) or not, given that our findings come from a sample of first year university students from different Chilean universities, although we are aware that the number is not large enough to consider that these findings can be generalized. For this reason, we plan to increase this sample in future studies. Regarding the analyses, future studies could use machine learning algorithms to evaluate the robustness of the results, for example, k-fold cross validation.

Regarding the dropout intention construct, it is necessary to draw attention to the way it is measured, an issue in which there is still a long way to go psychometrically. A systematic review on dropout intention in university students showed that there are few investigations that employ scales with adequate psychometric properties for the measurement of dropout intention (Sáez-Delgado et al., 2019). Another research (Meyer et al., 2022) found three aspects that could explain career change in first-year students: individual achievement in secondary education, a (mis)fit between individual occupational interests and study contents, and the social expectations of parents and peers regarding initial subject choice. It is suggested to pay special attention to the type of dropout intention that is intended to be measured. Conceptually, the intention to drop out can be considered as a general construct that accounts for the intentions to leave the university definitively; a more specific analysis involves specifying whether the student is thinking of leaving the university for a while and then returning or abandoning his or her career and studying at another university, or whether he or she wants to study another major at the same university. On the other hand, the analysis of critical variables in the first year, such as academic performance, could be useful. Previous literature shows that complete university dropout and change to another university or major program underlie different decision-making processes.

## 5. Conclusion

The present study findings and the above discussion yield the following conclusions: (a) motivational-cognitive variables are an effective channel for understanding the phenomenon of dropout intention, as it could be modified in the teacher-student interaction, the study did not show that the teacher-student interaction was modified; (b) the obtained model showed that 38.9% of the variance of dropout intention and 27.4% of the variance of career satisfaction are due to predictor variables: perceived social support, social self-efficacy, academic purposes, university social satisfaction, and perceived academic performance; (c) social self-efficacy favors social satisfaction at university, which, in turn, positively impacts on academic satisfaction and low dropout intention; (d) university social satisfaction is the most important mediating variable in relations of social self-efficacy, academic purposes, career satisfaction and dropout intention; (e) the perception of academic performance has less influence on dropout intention and on career satisfaction among first-year students; and (f) academic purposes are, to a large extent, association of dropout intention.

In conclusion, it is a fact that raising the quality of teaching and learning processes requires proactive behavior on the part of teachers and students. This means that universities are required to design and develop teaching interventions that will enable educators to generate personal resources for promoting and supporting the variables, as this study has demonstrated, that impact critically on dropout intention, and, consequently, are key for preventing students’ definitive disengagement from university.

## Data availability statement

The original contributions presented in the study are included in the article/supplementary material, further inquiries can be directed to the corresponding author.

## Ethics statement

The studies involving human participants were reviewed and approved by the Ethics Committee of the Universidad de Concepción, Chile. The patients/participants provided their written informed consent to participate in this study.

## Author contributions

YL-A contributed to the literature systematic review, to the design, as well as the data extraction, data analysis, abstract, the writing of the manuscript, and full-text review. FS-D contributed to the design of the study, abstract, full-text review, and the writing of the manuscript. JM-N abstract, full-text review, and the writing of the manuscript. AB and AD-M contributed to the interpretation of the results, the writing of the manuscript, and full-text review. All authors contributed to the article and approved the submitted version.

## Funding

This work was supported by Doctoral Program of the Department of Psychology, Universidad de Concepción, CONICYT-PCHA/National Doctorate Grants/2017-21170795, VRID-Multidisciplinary Project N°2021000397MUL, Master’s Degree in Psychology and Vice-Rectory for Research and Development, and the FONDECYT Initiation Project N°11230864 entitled “Academic and life purposes, social adaptation, emotional, motivational, and academic self-regulation: A mixed design to explain dropout intention and university academic performance”.

## Conflict of interest

The authors declare that the research was conducted in the absence of any commercial or financial relationships that could be construed as a potential conflict of interest.

## Publisher’s note

All claims expressed in this article are solely those of the authors and do not necessarily represent those of their affiliated organizations, or those of the publisher, the editors and the reviewers. Any product that may be evaluated in this article, or claim that may be made by its manufacturer, is not guaranteed or endorsed by the publisher.
